# Multimodal Treatment of a Spontaneously Ruptured Echinococcus Cyst of the Spleen and Its Complications

**DOI:** 10.1155/crgm/6657981

**Published:** 2025-02-20

**Authors:** Sévérine De Bruijn, Annick De Weerdt, Glenn Broeckx, Maarten Spinhoven, Rudi De Paep, Dominique Robert, Niels Komen, Philippe G. Jorens

**Affiliations:** ^1^Department of Intensive Care Medicine, Antwerp University Hospital, University of Antwerp, Edegem, Belgium; ^2^Department of Hematology of the Antwerp University Hospital, University of Antwerp, Edegem, Belgium; ^3^Department of Pathology, Antwerp University Hospital, University Hospital of Antwerp, Edegem, Belgium; ^4^Department of Pathology of the ZNA Middelheim Hospital, Antwerp, Belgium; ^5^Department of Radiology, Antwerp University Hospital, University of Antwerp, Edegem, Belgium; ^6^Department of Abdominal and Reconstructive Surgery, Antwerp University Hospital, University of Antwerp, Edegem, Belgium; ^7^Antwerp ReSURG, Antwerp Surgical Training, Anatomy and Research Centre (ASTARC), Faculty of Medicine and Health Sciences, University of Antwerp, Wilrijk, Belgium

**Keywords:** continuous veno-venous hemofiltration with gradient sodium replacement, echinococcosis, hypernatremia, peritoneal lavage, splenic cyst rupture, splenic hydatidosis

## Abstract

**Introduction:** Cystic echinococcosis, also known as hydatid cyst, is a parasitic infection of mammals that can affect any organ. Although the diagnosis of primary splenic echinococcosis is challenging, especially in nonendemic areas, it can be life-saving because an anaphylactic shock may occur when the cyst ruptures. Recommendations regarding optimal treatment options after rupture are scarce, and the overall prognosis remains poor.

**Case Presentation:** A patient with a spontaneous rupture of an isolated splenic hydatid cyst was treated with splenectomy and peritoneal lavage with a hypertonic salt solution. The patient survived despite rapidly progressive hypernatremia, which was treated with conventional therapy along with continuous venovenous hemofiltration with gradient sodium replacement.

**Discussion:** When the decision is made to treat a patient with a spontaneously ruptured splenic echinococcus cyst, splenectomy is the only surgical option. Hypernatremia is a complication to be expected when hypertonic saline is used to rinse the splenic and abdominal cavities.

**Conclusion:** This case highlights the importance of prompt surgical intervention and the management of hypernatremia in patients with ruptured splenic hydatid cysts.

## 1. Introduction

Cystic echinococcosis, also known as hydatid cyst, is a parasitic infection caused by the larval stage of *Echinococcus granulosus*. This disease is a significant health problem in sheep- and cattle-raising countries worldwide, but it is seldom seen in Europe [1, 2]. However, due to migration, exresidents of regions where echinococcosis is endemic have relocated to areas with limited knowledge of this disease, posing a diagnostic and treatment challenge [3].

Echinococcosis is a zoonosis, with humans serving as accidental intermediate hosts [2, 4]. The infection can affect any organ, but it most frequently invades the liver and lungs [5]. Isolated splenic hydatidosis is a very rare form of hydatid disease, which usually remains asymptomatic until cyst rupture occurs. The rupture of a hydatid cyst, whether spontaneous or traumatic, can result in severe anaphylactic reactions and dissemination of the infection [6]. This makes early diagnosis and intervention crucial.

Recommendations for the optimal management of ruptured splenic hydatid cysts are limited, and the overall prognosis remains poor. Treatment options typically include surgical intervention, such as splenectomy, combined with anthelmintic therapy to prevent recurrence and manage complications [3, 7]. This case report presents a patient with a spontaneous rupture of an isolated splenic hydatid cyst, highlighting the importance of prompt surgical intervention and the management of hypernatremia in such cases.

## 2. Case Presentation

**Figure 1:**
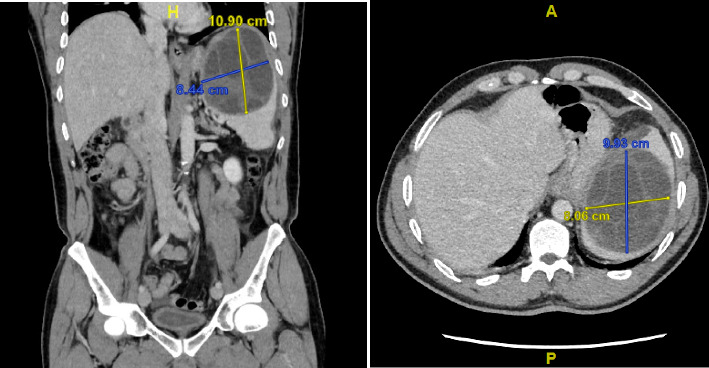
Coronal and axial CAT-images of the abdomen showing a cystic lesion in the spleen with septa and daughter cysts consistent with a splenic hydatid cyst.

A 56-year-old Macedonian man residing in Belgium for 13 years visited the emergency department because of acute onset epigastric pain. Clinical examination revealed a distended tender abdomen and a palpable mass in the left hypochondrium. Lab results showed a slightly elevated C-reactive protein (66.5 mg/L, normal value < 10 mg/L), while cytology and liver enzymes were completely normal. Abdominal computed axial tomography (CAT)-scan revealed a multiloculated cystic process in an enlarged spleen, with signs of anterior cranial extracapsular breakthrough (Figure 1).

Heteroanamnesis revealed that the patient's wife had undergone surgery for a hepatic hydatid cyst in the past. On account of a suspicion of a ruptured splenic hydatid cyst, anthelmintic therapy (albendazole and praziquantel) was initiated prior to surgery. The patient subsequently underwent an urgent laparotomy and splenectomy.

Gauzes drenched in hypertonic saline were placed around the spleen. After splenectomy, the entire abdominal cavity was rinsed with a 20% hypertonic saline solution. Maximum serum sodium concentration during surgery amounted to 161 mmol/L. Intravenous administration of 5% dextrose in water was commenced. Nevertheless, the serum sodium concentration reached 170 mmol/L on ICU admission, approximately 90 min after the first use of the hypertonic solution, quickly rising to 177 mmol/L. Despite the administration of large amounts of intravenous dextrose 5% in water, the serum sodium concentration only “dropped” to a mere 172 mmol/L, 8 h after admission. Continuous venovenous hemofiltration (CVVH) with gradient sodium replacement fluid was initiated, which resulted in a gradual decrease of the serum sodium concentration over days.

Thrombosis of the right superficial femoral artery, necessitating urgent thrombectomy, occurred 12 h after ICU admission. In addition, a left-sided pleural effusion, compromising ventilation, was drained via chest tube. The patient developed convulsions treated with phenytoin. Praziquantel administration was discontinued taking its proepileptogenic properties into account. Episodes of grand mal epilepsy continued to occur, necessitating additional antiepileptics (topiramate and levetiracetam). MRI of the brain approximately 1 week later showed areas of restricted diffusion interpreted as atypical presentations of extrapontine myelinolysis.

**Figure 2:**
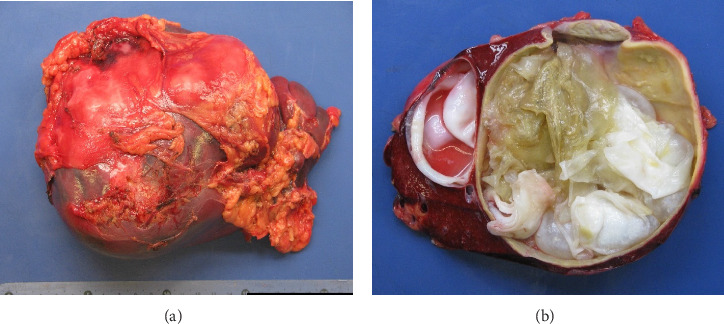
(a) Gross examination of the spleen, showing splenomegaly due to a multilocular cystic process. (b) The cysts are multilayered and contain a gelatinous laminar mass.

**Figure 3:**
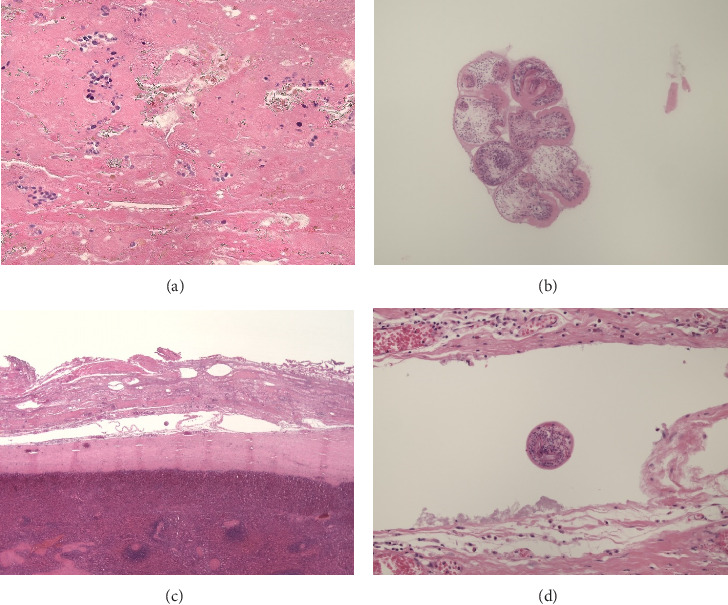
(a) The gelatinous laminar mass contains birefringent material. (b) Viable protoscolices are seen. (c) Overview of the wall of the cyst with an outer layer of fibrous tissue and an inner layer of laminar hyaline tissue. (d) In the wall of the cyst, viable protoscolices are found as well.

Ex vivo cleavage of the extirpated subcapsular splenic cyst (Figure 2(a)) revealed a multilocular process containing a gelatinous laminar mass. A two-layered cystic wall was found: the inner white layer was smooth and easily removable and the outer layer was firm and yellowish (Figure 2(b)). Microscopy of the cystic content revealed a fibrillary amorphous debris containing birefringent hooks, nuclei (Figure 3(a)), and viable protoscolices of *E. granulosus* (Figure 3(b)). The inner layer of the cystic wall contained lamellar hyaline membranes with viable protoscolices (Figures 3(c) and 3(d)); the outer layer consisted of dens fibrotic tissue with inflammation and calcification (Figure 3(c)).

Several complications, including a stomach perforation and ischemia and perforation of the ascending and transverse colon and small intestine, with subsequent secondary peritonitis, arose. The stomach perforation was sutured, a partial duodenectomy in combination with a subtotal colectomy was performed and caspofungin, and vancomycin were administered given the presence of *Candida glabrata* and *Enterococcus faecium* in the perioperatively prelevated abdominal fluid. Pulmonary empyema caused by *Candida glabrata* a month later was treated with caspofungin. Source control in the form of drainage during thoracotomy was performed. A perforation of the diaphragm was repaired. Four weeks later, CAT-guided puncture of an abscess in the splenic cavity was executed. This time *Candida albicans* was the causative pathogen. Treatment with fluconazole was initiated.

Renal replacement therapy remained necessary for a month. Albendazole therapy was continued (for 6 months) and antiepileptics were gradually tapered and discontinued. Approximately 6 weeks after admission, artificial ventilation was ceased, after which it became clear that a personality change (DD. ICU delirium/shock/sequelae extrapontine myelolysis) had taken place. Combination therapy with quetiapine (antipsychotic), trazodone (SNRI), and mirtazapine (tetracyclic antidepressant) were needed to suppress the patient's agitated state. The patient remained in the intensive care ward for a total duration of 66 days.

After ICU discharge, seizures reoccurred, personality changes persisted, and cognitive impairment became apparent. The patient remains in need of neuropsychiatric follow-up. There is, however, now 5 years after the presentation, not been any sign of recurrence of *Echinococcus granulosis* infection to this day.

## 3. Discussion

Hydatid disease, a faeco-orally transmitted parasitic infection, is caused by Taeniidae cestode parasites, with *Echinococcus granulosus* being most prevalent [2, 8, 9]. Dogs are usually the definitive host; sheep, goats, camels, pigs, and equines are intermediate hosts. Humans only act as an accidental host with no role in the transmission [2, 8, 10]. Most hydatid cysts are acquired in childhood. Today echinococcosis is spread worldwide due to changing patterns of emigration. In Europe, *Echinococcus granulosus* is most prevalent in animals living in Spain, Italy, Greece, Poland, and Slovakia; however, the parasite is not endemic in Belgium [11].

After ingestion, Echinococcus embryos (oncospheres) penetrate the intestinal mucosa, enter the blood or lymphatic system, and migrate to organs of the intermediate or accidental host [2, 10]. Isolated splenic hydatidosis is an extremely rare entity, as cyst embryos require primary infestation through the arterial route and are trapped in the liver or the lung, before they arrive in the splenic sinusoids [10]. Splenic echinococcosis can also arise due to retrograde spread through the portal and splenic veins in patients with portal hypertension or following systemic dissemination or intraperitoneal spread complicating rupture of a hepatic cyst [1, 6]. In our patient with a solitary splenic hydatid cyst, the etiopathogenesis of the arterial route is more likely as no previous hepatic echinococcosis had been reported and no hepatic or other organ echinococcosis was seen on CAT scan or during surgery.

Symptoms depend on the size and the anatomical location of the cyst [12]. The symptoms are usually nonspecific and mild such as abdominal discomfort, dyspepsia, constipation (compression of the colon), dyspnea (rise of the left diaphragm), and a palpable mass in the left upper quadrant (splenomegaly) [1, 13, 14]. Peritonitis, urticarial rashes, fever, and abdominal distension can be seen after hydatid rupture. In these patients, removal of a disrupted cyst can cause a potentially fatal anaphylactic shock [15–17]. Spontaneous rupture in the peritoneal cavity as a result of increased intracystic pressure is a rare complication but can occur in a subcapsular located splenic cyst as was the case in our patient [6].

Diagnosis of splenic hydatidosis is initially made by ultrasound (US) or CAT, showing cysts, calcification, and cavities. The presence of collapsed membranes in a cystic lesion is pathognomonic [16]. Eosinophilia is a common hematological finding but was absent in our patient [1]. Serologic screening tests that are frequently used are immunoglobulin G antibody detection in the blood using enzyme-linked immunosorbent assay and the indirect hemagglutination test (IHA) [18, 19]. In our patient, the IHA amounted to 1/320 with an ELISA test deemed positive with a ratio of 2.51. The diagnosis is usually confirmed by histopathological examination: (1) a thick, acellular, laminated layer with acidophilic staining, (2) a cellular germinal layer, and (3) the presence of brood capsules or protoscolices are pathognomonic and were all unquestionably present in our patient [20].

Data on optimal treatment for a ruptured splenic Echinococcus cyst are very scarce. An emergency splenectomy appears to be the golden standard [1, 6, 21]. As a ruptured cyst may release protoscolices and, therefore, cause secondary hydatidosis, radical emergency surgery removing all cystic contents needs to be followed by irrigation of the peritoneal cavity with a protoscolicidal agent (e.g., povidone iodine (10%), silver nitrate (0.5%), and hypertonic saline (3%–30%)) [6, 22–26]. In our patient, readily available hypertonic saline was chosen, given the urgency of the procedure and considering its powerful protoscolices inactivating capacity in vitro, in animal models and in humans during elective and emergency surgery despite the possible fatal complication of hypernatremia, which unfortunately arose. The rapid increase in serum sodium concentration can be held accountable for osmotic demyelination which led to the neurologic symptoms [27] and the thrombosis of the femoral artery can be attributed to a hypernatremia–hyperosmolality-induced hypercoagulability due to an increase in endothelial production and secretion of von Willebrand factor [28]. The gastrointestinal perforation observed in our patient may have been iatrogenic, potentially caused by the surgical procedures or the use of hypertonic saline. This unfortunate complication highlights the need for careful consideration of the risks associated with the use of hypertonic saline and the importance of meticulous surgical technique to minimize the risk of iatrogenic injuries. The novelty in our case report regards the use of CVVH with gradient sodium replacement to lower the serum sodium concentration after failure of conservative measures.

This case underscores the importance of prompt surgical intervention and the management of hypernatremia in patients with ruptured splenic hydatid cysts. Future cases should consider alternative methods for managing hypernatremia and minimizing the risk of gastrointestinal perforation.

## 4. Conclusion

When the decision is made to treat a patient with a spontaneously ruptured splenic echinococcus cyst, splenectomy is obviously the only surgical option. Given that hypernatremia is a likely complication associated with the use of hypertonic saline for irrigation of the splenic and abdominal cavities, we advise to take treatment with CVVH with gradient sodium replacement into account whenever hypernatremia in the peri- or postoperative period does not subside with conventional therapy.

## Data Availability

The data that support the findings of this study are available from the corresponding author upon reasonable request.
